# Hypertension in a low-income, predominantly afrocolombian city: Prevalence, awareness, treatment, control, and risk factors from a community-based screening

**DOI:** 10.7705/biomedica.7721

**Published:** 2025-09-22

**Authors:** Karen D. Palomino, Daniela Molano, Diego I. Lucumí, Yaicira Maturana

**Affiliations:** 1 Fundación Universitaria San Martín, Bogotá, D. C., Colombia Fundación Universitaria San Martín Bogotá D. C Colombia; 2 Facultad de Medicina, Universidad de los Andes, Bogotá, D. C., Colombia Universidad de los Andes Facultad de Medicina Universidad de los Andes Bogotá D. C Colombia; 3 Escuela de Gobierno Alberto Lleras Camargo, Universidad de los Andes, Bogotá, D. C., Colombia Universidad de los Andes Universidad de los Andes Bogotá D. C Colombia; 4 Secretaría Municipal de Salud de Quibdó, Alcaldía de Quibdó, Chocó, Colombia Alcaldía de Quibdó Chocó Colombia

**Keywords:** Noncommunicable diseases/prevention and control, hypertension, mass screening, prevalence, awareness, enfermedades no transmisibles/prevención y control, hipertensión, tamizaje masivo, prevalencia, concienciación

## Abstract

**Introduction.:**

Hypertension is a serious medical condition associated with high morbidity and mortality. The prevalence of hypertension is increasing in lower-middle-income countries, but the lack of local data can hinder the planning and development of strategies to manage this condition.

**Objectives.:**

To determine the prevalence, awareness, treatment, and control of hypertension in Quibdó, a predominantly afrocolombian middle-sized city. Additionally, we aimed to describe the distribution of risk factors and analyze the associations among clinical outcomes, demographic characteristics, behaviors, and prior conditions.

**Materials and methods.:**

This cross-sectional study used secondary data from a community-based screening conducted by local government institutions between May and September 2019.

**Results.:**

Among the participants screened, 892 (21%) had hypertension and 46.52% were aware of their diagnosis. Of the participants that were aware, 65.3% were receiving pharmacological treatment. However, only 54.61% of participants receiving treatment had controlled hypertension, meaning that only 16.5% of the population with hypertension had adequate awareness, treatment, and control. Additionally, 50.43% of the participants without hypertension had prehypertension and 62.53% of the population had excess body weight. While 91.81% had their blood pressure checked within the past year, there remains a persistent issue within the health care system.

**Conclusion.:**

The prevalence of hypertension in communities in lower-middle-income countries such as Quibdó is concerning, as is the low awareness, treatment, and control of this condition. Community-based screenings are useful; however, a gap remains in translating these efforts into effective public health prevention strategies and clinical practice. This highlights the need for future research to support the adoption of more comprehensive approaches to hypertension prevention and treatment in underserved communities.

## Resumen

**Introducción.:**

La hipertensión es una condición médica grave asociada con gran morbilidad y mortalidad. La prevalencia de hipertensión está aumentando en los países de ingresos medianos o bajos, pero la falta de datos locales puede obstaculizar la planificación y el desarrollo de estrategias para manejar esta condición.

**Objetivos.:**

Estimar la prevalencia, conocimientos, tratamiento y control de la hipertensión en Quibdó, una ciudad intermedia con población predominante afrocolombiana. Describir la distribución de los factores de riesgo y analizar la asociación entre los resultados clínicos, las características demográficas, los comportamientos y las condiciones previas.

**Materiales y métodos.:**

Este estudio de corte transversal utilizó datos secundarios de un tamizaje comunitario realizado por instituciones gubernamentales locales entre mayo y septiembre del 2019.

**Resultados.:**

Entre los evaluados, 892 (21 %) tenían hipertensión, y el 46,5 % conocía su diagnóstico. De quienes lo conocían, 65,3 % recibía tratamiento farmacológico. Sin embargo, solo el 54,6 % de los tratados estaban controlados, lo que significa que apenas el 16,5 % de la población con hipertensión tenía conocimiento, tratamiento y control adecuados. Además, el 50,4 % de los participantes no hipertensos presentaba prehipertensión y el 62,5 % de la población tenía exceso de peso. Aunque al 91,8 % se le había medido la presión arterial en el último año, persiste un problema dentro del sistema de salud.

**Conclusiones.:**

La prevalencia de hipertensión en comunidades de países de ingresos medianos o bajos, como Quibdó, es preocupante, al igual que los bajos niveles de conciencia, tratamiento y control. Los tamizajes comunitarios son útiles; sin embargo, persiste una brecha en transformar estos esfuerzos en estrategias efectivas de prevención en salud pública y en la práctica clínica. Esto resalta la necesidad de futuras investigaciones que sustenten la adopción de enfoques más integrales para la prevención y el control de la hipertensión en contextos desatendidos.

Hypertension is a serious and silent medical condition associated with a high burden of morbidity and mortality, especially attributed to an increased risk of cardiovascular disease and its complications ^1^. According to the World Health Organization (WHO), 1.28 billion adults aged 30-79 years have hypertension ^1^. Current data indicate that nearly two-thirds of hypertensive adults reside in low- and middle-income countries ^2^.

Although the global prevalence of hypertension is increasing, it has not been uniform across regions. A more pronounced increase in the adult hypertensive population has been observed in low- and middle-income countries ^3^, primarily driven by an increase in hypertension-related risk factors, including unhealthy diets (excessive salt consumption and low intake of fruits and vegetables), physical inactivity, excessive alcohol consumption, and excess body weight ^4^.

Compared with high-income countries, low- and middle-income countries in Latin America have historically lacked comprehensive data on hypertension. However, recent studies and meta-analyses have helped to narrow this gap, revealing a consistently high burden of both hypertension and prehypertension in the region.

The updated CARMELA study reported that 42.5% of adults had hypertension (46.6% of men and 38.7% of women), and 32.5% had prehypertension (36.0% of men and 29.4% of women). While 63.0% of hypertensive individuals were aware of their condition, only 48.7% were receiving pharmacological treatment and 21.1% achieved blood pressure control ^5^. These results are supported by a systematic review and metaanalysis conducted by Vera-Ponce *et al.,* which confirmed the widespread prevalence of prehypertension across Latin America. Similarly, Lamelas *et al.* reported significant disparities in the awareness and control of hypertension between rural and urban populations ^6^^,^^7^. Collectively, these findings underscore the need for tailored, community-based public health strategies aimed at improving the diagnosis, treatment, and control of hypertension in Latin America's diverse and underserved populations.

In Colombia, national surveys provide additional insight into the prevalence of hypertension among older adults. According to the Colombian SABE study *(Salud, Bienestar y Envejecimiento),* which evaluated adults 60 years and older, the overall prevalence of hypertension was 60.2%. Importantly, this study examined disparities in prevalence based on skin color, categorized as light, medium, or dark. Among men, the prevalence of hypertension was 53.2% for participants with light skin, 49.6% for participants with medium skin, and 49.4% for participants with dark skin. Among women, the prevalence of hypertension increased across skin tones, with 62.5% for participants with light skin, 61.7% for participants with medium skin, and, notably, 69.9% for participants with dark skin ^8^. These results highlight significant disparities in hypertension that may reflect the impact of social and structural factors.

Similarly, the Colombian PURE study, a prospective population-based cohort study including 3,745 adults from three ethnic groups (552 white, 2,746 mestizo, and 447 afro-descendant), reported an overall hypertension prevalence of 39.2%. Afro-descendant participants had the highest prevalence at 46.3%, followed by white participants at 41.5% and mestizo participants at 37.6%. Notably, the higher prevalence among afro-descendant participants was largely driven by afro-descendant women. The study revealed that hypertension was consistently associated with older age, higher body mass index, waist circumference, and waist-to-hip ratio across all ethnic groups.

Moreover, low educational attainment was strongly associated with hypertension, particularly among afro-descendant participants, 70% of whom had low levels of education compared to 52% of white participants. Only 7% of afro-descendant participants had a university education, whereas 26% of white participants did. Although education itself is not a direct causal factor, it serves as a proxy for socioeconomic status, which has emerged as a key determinant of hypertension prevalence ^9^. These findings underscore the role of social inequities in shaping ethnic disparities in cardiovascular health in Colombia.

According to the Colombian fund for high-cost diseases, from July 1^st.^, 2019, to June 30^th.^, 2020, the prevalence of hypertension in Colombia was 9.08 cases per 100 inhabitants. Remarkably, the data underestimates the prevalence since it is only for people who are treated in the health system ^10^ . Another study was conducted by extracting and analyzing data from SISPRO, a dataset of the *Ministerio de Salud y Protección Social* of Colombia that includes individual records of health services provision (RIPS by its acronym in Spanish). In this study, the prevalence of arterial hypertension was calculated and standardized by age and for the different geographic regions of the country from 2013 until 2017. During the five years evaluated, the average national prevalence for individuals over 60 years was 28.14% ^11^. However, the authors suggested that one of the study's limitations was underreporting in the data.

While the prevalence of hypertension is increasing in low- and middle-income countries, the paucity of local data can hinder the planning and development of preventive and clinical care strategies to manage this chronic condition. In this sense, local data has the potential to increase political will, as it might inspire social and political leaders to pursue solutions for neglected public health problems ^12^. In Colombia, local population health data are needed, particularly in disadvantaged middle-sized urban areas ^13^. These areas may be especially vulnerable to factors that shape the development of hypertension and disparities such as poverty, income inequality, and the armed conflict in Colombia that has resulted in one of the world's largest internally displaced populations ^14^.

To improve the availability and accuracy of local data for hypertension in disadvantage middle-sized populations, a community-based screening was carried out in Quibdó, the capital of the department of Chocó and a middle-sized Colombian city.

Quibdó faces numerous social, political, and economic challenges. In 2019, the year in which this study was conducted, the population of Quibdó was 130,042, of which 86.7% were urban dwellers, 92.7% were afro-Colombians, and the remaining 7.3% were indigenous and mestizo people ^15^. The same year, 65.6% of the population lived in poverty ^16^ and the city had one of the highest unemployment rates in Colombia at 20.1%, compared with the national average of 10.8% ^15^. In 2019, the city also struggled with inadequate urban planning, poor urban planning, and a high rate of homicide (77.4 per 100,000 inhabitants) ^17^. These disadvantages may increase the risk of hypertension and limit access to health care services ^18^, which is further exacerbated by the scarcity of health records for hypertension in the overall general population.

These distinctive characteristics of Quibdó provide a pivotal contextual foundation, emphasizing the importance of considering the distinct experiences and health dynamics of its predominantly afrocolombian population. A study that reflects these contextual dynamics in marginalized urban contexts is essential for addressing the specific demands and challenges faced by afrocolombians in the prevention and management of hypertension.

This study had three objectives. First, to estimate the prevalence of hypertension and its awareness, treatment, and control through a community-based screening carried out in Quibdó. Second, to describe the distribution of risk factors for hypertension and health-related behaviors within the population screened. Finally, to estimate the associations between clinical endpoints and demographic characteristics, behaviors, and preceding conditions with systolic blood pressure and hypertension using a multivariate approach.

## Materials and methods

### Study design

This was a cross-sectional study based on secondary data collected through a community-based screening in rural and urban areas by two city government institutions *(Departamento de Salud* and *Departamento de Cultura, Recreación y Deporte)* between May and September 2019. This secondary data analysis was approved by the Universidad de los Andes IRB.

### Study population

The participants were recruited through health promotion campaigns that took place in schools and community centers in different areas of Quibdó. Locals who attended these campaigns were informed about the community-based screening and willing individuals who were 18 years or older were enrolled. Pregnant women were excluded. Furthermore, key risk factors for hypertension, such as family history, history of preeclampsia, and low birth weight, were not collected.

A formal sample size calculation was not performed to estimate the prevalence of hypertension, as the study was not designed with probabilistic sampling. Instead, similarly to other studies in this field, it was calculated based on an open community screening strategy ^19^^,^^20^. Therefore, all individuals who voluntarily attended the screening events and met the inclusion criteria were enrolled, making this a convenience (opportunistic) sample.

### Assessment tools

Prior to data collection, health care personnel -including nurse assistants and nursing students- received training to ensure standardization of blood pressure measurement procedures. Although no formal pilot study was conducted, all personnel involved were trained under professional nurse supervision.

Blood pressure was measured with adequately calibrated automatic Omron™ sphygmomanometers while participants were at rest and seated. Measurements were taken two to three times, one to two minutes apart. In cases with three readings, the first was discarded, and the average of the second and third measurements was used for analysis, which is consistent with the finding that the first blood pressure reading is often higher than subsequent ones ^21^. Participants with fewer than two valid readings were excluded from the analysis. However, we were unable to ensure that participants avoided consuming stimulant beverages (such as coffee, energy drinks, or sugar beverages) 30 minutes prior to obtaining blood pressure measurements or that they had an empty bladder at the time of the reading.

Data regarding previous hypertension diagnosis, treatment, demographic characteristics, risk factors for hypertension, and health-related behaviors were obtained by the same trained assistants through three sets of questionnaires. Each entity had its own questionnaire with different questions and lengths according to the financial resources available, leading to variation in question format, length, and completeness across participants. This explains the difference in the amount of data collected for each variable of interest. Verbal consent was obtained from all the participants.

### Variables

The dependent variables were systolic blood pressure, using the average of the second and third measurements, and hypertension (average of the last two readings of systolic blood pressure ≥ 140 mm Hg or diastolic blood pressure ≥ 90 mm Hg or taking antihypertensive medication).

In addition, we created variables for awareness (participants with hypertension who had previous knowledge of this diagnosis), treatment (participants with hypertension who were taking medication), control (participants who were receiving treatment and had values for systolic and diastolic blood pressure under 140 mm Hg and 90 mm Hg, respectively), and prehypertension (defined as a systolic blood pressure between 120 and 139 mm Hg and/or diastolic blood pressure between 80 and 89 mm Hg).

The covariates were race (afrocolombians, mixed and indigenous people), body mass index (BMI), excess body weight (defined as a BMI greater than 25 kg/m^2^), alcohol consumption (never or almost never, 1-3 times per week, more than 3 times per week), smoking (yes or no), and preceding conditions such as diabetes mellitus diagnosis (yes or no), acute myocardial infarction (yes or no), and stroke (yes or no). A morbidity index was created by adding the associated morbidity factors, such as the diagnosis of diabetes mellitus, acute myocardial infarction, and stroke. This index has a score from 0 to 3. All analyses were adjusted for age (continuous) and sex (men or women).

### Statistical analysis

Statistical analysis was performed in Stata SE 17.0™. Regarding the descriptive analysis, absolute and relative frequencies were calculated for the qualitative variables. Measures of central tendency were estimated for the quantitative variables. Proportions were calculated for participants with hypertension, awareness, treatment, control, and prehypertension.

We conducted both linear and logistic regressions for continuous and binary dependent variables, respectively. Linear regression was carried out to assess the associations between systolic blood pressure, health-related behaviors and preceding conditions. Similarly, we ran a logistic regression to assess the associations between hypertension and covariates. Coefficients and odds ratios with 95% confidence intervals and p values were calculated for linear and logistic regressions, respectively. For these regressions, we restricted the sample to participants who had gender and age information available.

## Results

The total number of participants was 5,358. Because data collection was incomplete for some of the variables, the totals used in the different analyses varied. In total, 95.99% of participants were afrocolombian, 0.84% were mixed race (mestizos), 2.45% were indigenous and 0.17% were white. More women than men were screened; 17.3% of the participants were women, 14.25% were men, and there was no gender data available for the remaining participants. Eighty-two percent of the participants lived in urban areas. Ages ranged from 18 to 64 years, with a mean age of 34.91 years. With respect to health behaviors, almost 1 out of every 10 participants admitted drinking alcohol three or more days per week. Additionally, only 745 of 4,840 reported fruit consumption four or more days per week (table 1).

**Figure 1:**
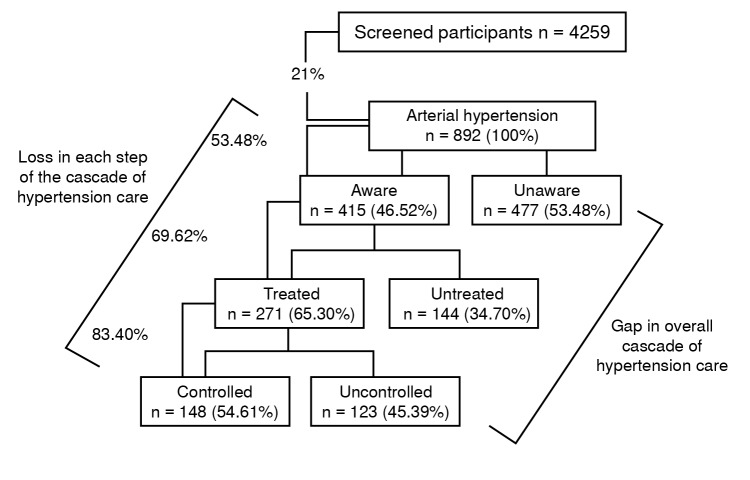
Proportions of hypertension, awareness, treatment, and control in a community-based screening in Quibdó, 2019

Of all the participants, 4,259 had second and third systolic and diastolic blood pressure measurements available and were considered screened. Among those, 892 (21%) individuals had hypertension, and 1,698 (50.43%) of the participants without hypertension had prehypertension. Figure 1 shows the prevalence, awareness, treatment, and control of hypertension. The data show that only 16% of the population screened for hypertension had adequate awareness, treatment and control of blood pressure. Table 1 shows the prevalence of prehypertension.

**Table 1:** Demographics, comorbidities, and health-related behavior

Variables		n	% / mean (SD)
Hypertension			
	Yes	892	20.9
	No	3,367	79.0
	Total	4,259	100
Prehypertension			
	Yes	1,698	50.4
	No	1,669	49.5
	Total	3,367	100
Systolic blood pressure		4,259	121.1 (17.31)
Diastolic blood pressure		4,259	75.7 (12.27)
Age		1,693	34.9 (13.25)
Race			
	White	38	0.7
	Indigenous	131	2.4
	Mixed	45	0.8
	Afrocolombian	5,126	9.9
	Total	5,340	100
Sex			
	Female	927	54.8
	Male	764	45.1
	Total	1,691	100
Area of residence			
	Rural	956	17.8
	Urban	4,402	82.1
	Total	5,358	100
Alcohol consumption			
	Never/almost never	3,175	59.41
	1 to 3 times a week	1,771	33.13
	More than 3 times a week	398	7.44
	Total	5,344	100
Active smoking			
	Yes	325	6.07
	No	5,021	93.92
	Total	5,346	100
Last blood pressure measurement			
	Less than a year ago	4,446	82.97
	Over a year ago	493	9.2
	Does not answer	14	0.26
	Does not know	72	1.34
	Never	333	6.21
	Total	5,358	100
Added salt			
	Never	1,718	35.81
	Seldom	701	14.61
	Sometimes	534	11.12
	Often	1,014	21.13
	Always	831	17.31
	Total	4,798	100
Physical activity in the last 7 days			
	Yes	1,048	96.14
	No	42	3.85
	Total	1,090	100
Fruit consumption per week			
	1 to 3 days	4,095	84.60
	4 to 5 days	472	9.75
	More than 5 days	273	5.64
	Total	4840	100
Vegetable consumption per week			
	1 to 3 days	2840	58.67
	4 to 5 days	415	8.57
	More than 5 days	1585	32.74
	Total	4840	100
Diabetes diagnosis			
	Yes	428	8.14
	No	4,824	91.85
	Total	5252	100
Myocardial infarction			
	Yes	49	0.91
	No	5296	99.08
	Total	5345	100
Stroke			
	Yes	25	0.4
	No	5,319	99.5
	Total	5,344	100
Morbidity index (diabetes mellitus, myocardial infarction, and stroke)			
	0 of the factors above	4,767	91.0
	1 of the factors above	449	8.5
	2 of the factors above	15	0.2
	3 of the factors above	5	0.1
	Total	5,236	100
BMI^a^		3,165	27.7 (5.8)
Excess body weight (BMI ≥ 25)			
	Yes	1,979	62.5
	No	1,186	37.4

**Figure 2:**
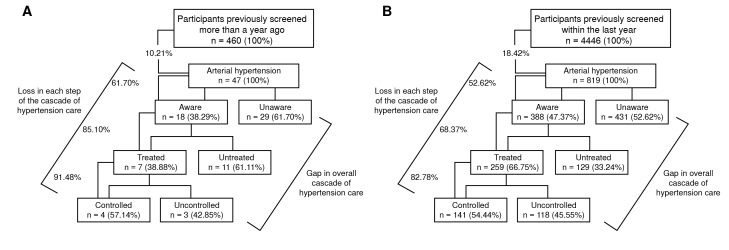
A. Proportions of hypertension, awareness, treatment, and control among previously screened participants more than one year prior. B. Proportions of hypertension, awareness, treatment, and control among previously screened participants within the last year.

Remarkably, 4,446 participants had their last blood pressure measurement taken less than one year prior. The prevalence, awareness, treatment, and control of hypertension for this group are shown in figure 2.

In table 2, we present factors associated with systolic blood pressure values below 140 mm Hg. In the multivariate linear regression analysis, we found statistically significant associations between systolic blood pressure and gender, age, and BMI.

In the multivariate logistic regression analysis (table 3), we found a statistically significant association for gender (women were less likely to have hypertension than men), age (younger individuals were less likely to have hypertension than older individuals), and BMI (individuals with lower BMI values were less likely to have hypertension).

Importantly, both tables include individuals with comorbidities and/or clinical outcomes linked to hypertension. In people with such conditions, the interpretation of blood pressure levels differs.

**Table 2:** Factors associated with systolic blood pressure values

		Systolic blood pressure					
		Bivariate model			Multivariate model		
Variables		Coef.	CI 95%		Coef.	CI 95%	
Age		0.25	0.18	0.36	0.22	0.13	0.35
Sex							
	Women	-9.7	-12.3	-7.21	-12.24	-14.80	-9.70
BMI		0.40	0.28	0.52	0.84	0.60	1.07
Alcohol consumption							
	0	Ref.			Ref.		
	1	-0.17	-1.30	0.95	-0.22	-2.85	2.40
	2	-0.88	-3.50	1.72	-1.73	-5.76	2.29
Currently smoking							
	No	Ref.			Ref.		
	Yes	2.76	0.62	4.90	5.47	-1.10	12.13
Comorbidity index							
	0	Ref.			Ref.		
	1	7.15	5.27	9.02	-0.5	-5.06	4.06
	2	4.25	-4.47	12.98	-6.33	-20.50	7.90

Linearity link test for all the models, p < 0.0001

**Table 3:** Factors associated with the presence of hypertension

		Hypertension					
		Bivariate model			Multivariate model if		
Variables		Odds ratio	CI 95%		Odds ratio	CI 95%	
Age		1.06	1.04	1.08	1.06	1.04	1.07
Sex							
	Men	Ref.			Ref.		
	Women	0.66	0.45	0.98	0.44	0.27	0.72
	BMI	1.03	1.02	1.05	1.10	1.05	1.14
Alcohol consumption							
	Never/almost never	Ref.			Ref.		
	1 to 3 times a week	0.70	0.59	0.83	0.86	0.53	1.40
	> 3 times a week	0.68	0.46	1.022	0.90	0.45	1.91
Currently smoking							
	No	Ref.			Ref.		
	Yes	1.43	1.08	1.90	1.50	0.49	4.61
Comorbidity index							
	0	Ref.			Ref.		
	1	2.49	1.98	3.13	0.69	0.31	1.55
	2	8.39	2.85	24.60	1.01	0.08	13.62

Linearity link test for all the models, p < 0.0001

## Discussion

This study was conducted with the purpose of estimating the prevalence, awareness, treatment, and control of hypertension; describing the distribution of risk factors for hypertension and health-related behaviors in the participants; and analyzing the associations between clinical endpoints and demographic characteristics, behaviors, and preceding conditions. One-fifth of the population had hypertension, with evidence of low rates of awareness, treatment and control. In the multivariate analysis, old age, male sex, and high BMI were associated with higher values of systolic blood pressure and a higher prevalence of hypertension.

Among residents of Quibdó, the prevalence of hypertension (21%) was lower than that reported in previous studies from other low- and middle-income countries ^22^^-^^24^, which can be explained by the fact that our population was younger than that reported in other studies, with a mean age of 34.91 years. An additional explanation is the greater participation of women (9% more than men), since women of fertile age seem to have lower systolic blood pressure values ^25^^,^^26^. The results of the multivariate analysis indicated that being female was associated with lower systolic blood pressure and a lower prevalence of hypertension. Previous studies suggest that female sex hormones may contribute to this protective effect by reducing the risk of elevated blood pressure ^27^.

The prevalence of hypertension is still a cause for concern, especially as there are two factors that place this community at a greater risk for this condition. First, with respect to the prevalence of prehypertension and the potential risk of progression to hypertension ^28^^,^^29^, only 49.57% of the individuals who were not hypertensive had normal blood values. This is particularly relevant for black individuals, considering that previous studies have shown that they have a 35% higher risk of transitioning from prehypertension to hypertension than white individuals ^30^.

The finding of racial differences in the progression from prehypertension to hypertension does not imply biological differences but might be explained by sociocultural and economic factors, including level of education, annual household income, neighborhood socioeconomic score, high poverty at the neighborhood level, lack of health insurance, and living in an area with a shortage of health professionals ^31^^,^^32^. Second, a high proportion of individuals with available data had excesss body weight (62.53%). Excesss body weight is a known factor related to hypertension incidence ^33^. For Colombia in general, this percentage corresponds to 56.4% ^34^.

In this population, undiagnosed hypertension is a common problem, with only approximately 1 out of 2 individuals with hypertension having previous awareness of their diagnosis. In a study conducted in 2019, that included 23,694 adults aged 60 years or older, 93.5% of black men and 95.7% of black women were found to be aware of their hypertension ^35^. The higher level of hypertension awareness observed among the population of black people in this study, compared with our target population, is likely attributable to age differences between the two groups. The black population of people in this study consists of individuals aged 60 years or older, whereas our target population is considerably younger, with a mean age of 34.91 years.

Importantly, among the 892 participants diagnosed with hypertension, 819 had their blood pressure assessed within the preceding year, highlighting a persistent issue within the health care system. Regrettably, there appears to be a gap in the translation of screening outcomes into effective interventions. This is particularly concerning given the predominance of the afrocolombian population in our study, for whom ineffective interventions -compounded by structural inequalities- continue to perpetuate disparities in health, disease experiences, and outcomes.

The rates of treatment (65.3%) and control (54.61%) in our study were higher than those reported in other studies conducted in low middle-income countries ^22^^,^^23^^,^^36^. This continues to be insufficient and could be partially attributed to limited access to routine clinical check-ups, clinical inertia, and the lack of referral to complementary care programs such as nutritional counseling and physical activity support. These challenges have been identified by the health authorities of Quibdó in the *Análisis de la Situación de Salud* (ASIS) via the model of social determinants of health ^37^. This model emphasizes that health outcomes are shaped not only by clinical care but also by broader structural and intermediate factors such as poverty, inequality, poor infrastructure, and weak institutional coordination. In Quibdó, these systemic barriers contribute to the inadequate delivery of health services and limited implementation of preventive programs, ultimately exacerbating the city's already fragile public health situation.

With respect to health behaviors within this community, a notable concern is the proportion of individuals who consume alcohol three or more days per week. We recommend implementing interventions aimed at reducing alcohol intake, as evidence suggests that such measures are effective in lowering blood pressure among both hypertensive and normotensive individuals and may also play a role in preventing the onset of hypertension ^38^.

Additionally, given the low proportion of individuals consuming fruits four or more days per week, we recommend efforts to improve access to this food group and to promote its daily consumption. Evidence indicates an inverse association between fruit intake and hypertension risk, with a daily intake of 100 g of fruit linked to a reduced risk of developing hypertension ^39^. Educational initiatives are essential to raise awareness of the health benefits of fruit consumption and to provide practical guidance for incorporating fruit into daily diets. From a social perspective, logistical barriers such as limited access to refrigeration or grocery stores can hinder the regular consumption of fresh fruits. To address these challenges, a comprehensive strategy could include public health campaigns, nutrition education, subsidies or incentives for fruit purchases, and policies aimed at improving access to fresh produce and encouraging healthier dietary behaviors.

Finally, increasing age and BMI were significantly associated with a higher prevalence of hypertension and higher systolic blood pressure values. We recommend giving BMI a central place in interventions, as it is a modifiable factor that has strategic importance since its management impacts multiple health outcomes.

These findings underscore the urgency of integrating blood pressure screening with patient education, ensuring timely referrals for individuals with elevated readings to health care professionals. These professionals can guide patients to adopt blood pressure-lowering behaviors, provide appropriate treatment, and facilitate access to regular follow-up care. Addressing structural barriers that disproportionately affect black populations is essential for improving their overall health outcomes. Previous studies have demonstrated that blood pressure screening campaigns using convenience samples -such as those employed in our study- are effective and relatively low-cost tools for increasing awareness of hypertension within the general population and potentially among health policymakers ^35^. Therefore, we recommend the continuation of such initiatives, particularly in resource-limited settings such as Quibdó.

Several limitations must be carefully considered when these results are interpreted. First, as this was a secondary data analysis, we had no control over the data collection process. The dataset was compiled from two sources, each employing different questionnaires and methodologies. This led to inconsistencies in data availability across key variables and the complete absence of certain important risk factors for hypertension, such as family history of hypertension, history of preeclampsia or eclampsia, and low birth weight. The absence of these variables may introduce residual confounding or bias in the estimation of associations.

Second, sample design and population distribution introduce potential biases. Although the study involved a substantial number of participants, the sample was drawn from a convenience-based screening campaign, which is not representative of the general population. This non-probabilistic sampling design may result in selection bias, particularly if the participants differ systematically from those who did not attend the campaign (e.g., in health awareness, access to care, or socioeconomic status).

Third, although we intended to conduct subgroup analyses, the uneven ethnic distribution of participants limited the statistical power of these comparisons. Most participants self-identified as afrodescendants, which reduced the precision of estimates for other ethnic groups and may have obscured potential differences in hypertension prevalence across populations.

Fourth, missing data posed an additional challenge. Although regression analyses were restricted to participants with complete datasets to reduce bias, this approach may still affect the generalizability of our findings. Participants with incomplete data may systematically differ from those included in the analysis, potentially skewing the results.

Fifth, this type of community screening for hypertension is typically cross-sectional by nature and hence limited in its ability to identify potential causal associations.

Finally, conducting research on low-resource settings such as Quibdó requires partnerships with government entities. While these collaborations enabled access to data, the lack of academic involvement in the design and implementation of data collection contributed to the methodological limitations observed. We strongly recommend strengthening institutional partnerships between academic researchers and public health authorities to increase data quality and ensure that future studies are methodologically rigorous, comprehensive, and capable of informing effective health interventions.

In conclusion, the prevalence of hypertension in rural communities in low-and middle-income countries such as Quibdó, Chocó, remains a significant public health concern. Equally troubling are the low levels of awareness, treatment, and control of this condition, as well as the difficulties in translating screening outcomes into effective interventions. These findings underscore the urgent need for the implementation of evidence-based interventions aimed at improving hypertension detection and establishing a cardiovascular care pathway centered on a healthy diet and physical activity, while also accounting for the influence of social determinants of health.

Future research should continue to support community-based screening initiatives, given their utility. Moreover, fostering continuous feedback among communities, health care providers, academic institutions, and policymakers is essential to generate actionable insights and inform the development of policies that effectively address the burden of hypertension.
